# Low-frequency repetitive transcranial magnetic stimulation for adolescent treatment resistant depression - a feasibility study

**DOI:** 10.1186/s12888-025-07115-5

**Published:** 2025-07-03

**Authors:** Jonas Jester-Broms, Helena Strömbergsson, Jonas Persson, Robert Bodén

**Affiliations:** Department of Medical Sciences, Uppsala University, Uppsala University Hospital, Uppsala, Sweden

**Keywords:** rTMS, Non-invasive brain-stimulation, Right frontal lobe, Youth, Teenagers, Depressive disorders

## Abstract

Depression is a debilitating mental health disorder that affects a significant portion of the adolescent population, and 20% of affected youths do not respond to two trials of treatment with antidepressants, i.e. suffer from treatment resistant depression (TRD). Repetitive transcranial magnetic stimulation (rTMS) has demonstrated efficacy in TRD in adults, but its application in the adolescent population remains relatively unexplored. This open label pilot study investigates the safety, feasibility, and potential efficacy of low-frequency (1 Hz) rTMS over the right dorsolateral prefrontal cortex (DLPFC) for adolescent TRD. Twenty patients, 13–18 years old, with treatment resistant unipolar- or bipolar depression were included (inclusion rate 0.9 patients per month) and two dropped out resulting in 18 participants completing the study (attrition rate 0.10). Daily sessions on 20–30 consecutive days with 1 Hz rTMS was administered. A significant decrease in depressive symptoms (decrease from median score of 35 to 23 on Montgomery-Asberg Depression Rating Scale, *p* < 0.01) and increase in function measures (increase of median score of 45 to 55 on Children’s Global Assessment Scale, *p* < 0.05) was observed at the end of treatment compared to baseline. However, only one patient reached the response criterium (< 50% score in Montgomery-Asberg Depression Rating Scale). Adverse effects such as scalp pain, headache, tiredness and nausea were common but tolerable, and tended to decrease during the course of treatment. In conclusion, the results of this study support feasibility of future larger studies of right frontal low-frequency rTMS for TRD in the adolescent population.

**Supplementary Information:**

The online version contains supplementary material available at 10.1186/s12888-025-07115-5.

## Introduction

Major depressive disorder (MDD) is now one of the leading causes of disability worldwide [1]. The first onset of MDD frequently occurs during adolescence and has a life-time prevalence of 12.9% worldwide [2]. MDD is one of the most common psychiatric disorders in childhood and adolescence. In Sweden, the adolescent lifetime prevalence of MDD has recently been estimated to be 11.4%, of which 22% reported to suffer from serious suicidal ideations [3]. Depression early in life has been associated with impaired school performances, interpersonal difficulties, increased risk of substance-use disorder, and other mental health disorders. Moreover, early onset of MDD is often recurrent and persists into adulthood which increases the risk for adverse outcomes later in life [4]. Treatment resistant depression (TRD) among adolescents has by some been defined as those who fail to respond to psychotherapy and 12 weeks of treatment with one selective serotonin reuptake inhibitor (SSRI) [5] while others propose a failure to respond to two antidepressants [6]. Approximately 20% of youths with depression fail to respond to two trials of SSRI and there is no consensus guideline for how to treat those patients [7].

Repetitive Transcranial Magnetic Stimulation (rTMS) is an increasingly used therapeutic option for TRD in adults with mild side effects, high tolerability, and administered with the patient being awake [8–10]. The mechanism of action has not been fully clarified but rTMS induces local electrical currents in the stimulated neurons and has been shown to induce long-term inhibition or excitation of groups of neurons in treated cortical areas [11].

Currently, the most robust evidence for rTMS as a treatment for psychiatric disorders has been shown for unipolar TRD although studies also report positive outcomes in bipolar depression [12].

In adolescents, high-frequency left-sided rTMS has been the most commonly applied protocol to investigate the treatment effect for unipolar TRD. All but two studies [13, 14], have been small open label studies and case-series. Croarkin et al., performed a multi-centre, double-blind study on rTMS treatment of adolescent TRD. In that study, 10 Hz rTMS was applied on the left DLPFC and no significant difference in depressive symptoms between active and sham stimulation was observed. In a recent three-armed Chinese study of first-episode depression, both 10 Hz rTMS left and 1 Hz over the right DLPFC was more effective than sham stimulation. This is the only currently available sham-controlled randomized trial to support the efficacy of high-frequency stimulation of the left DLPFC. Another, shorter 1 Hz protocol over the right DLPFC has also been used successfully among adult patients [9, 15], and this protocol has been reported to be more tolerable and to have a much lower risk of inducing seizures, which makes it a suitable candidate for further exploration in adolescents with depression. Indeed, five randomized controlled studies have investigated low frequency rTMS with mixed but promising results in adolescent patients with first-episode treatment naïve depression [14, 16–20], and one study reported 50% response rate in drug-free adolescent patients with depression where pharmacological resistance was not assessed [21]. However to our knowledge, there are no previous studies that investigate low-frequency rTMS in an adolescent TRD population, specifically.

The aim of this open label pilot study was to investigate the feasibility and safety of 1 Hz right hemisphere rTMS treatment in adolescent TRD as well as its potential antidepressive efficacy in this understudied population.

## Materials and methods

### Clinical trial registration

The trial was registered with the Clinical Trials Registry (identifier NCT06126198, registration date 2023-11-09, https://clinicaltrials.gov) and assigned a local trial identifier Teen-TMS_UU2023.

### Patient population

The patients were enrolled by referral from their regular psychiatric care facilities. At the screening visit, the patients were assessed regarding inclusion and exclusion criteria. Inclusion criteria were: informed consent from parents and legal guardian, age 13–19 years, diagnosis of uni- or bipolar depression verified through a Mini International Neuropsychiatric Interview for children and adolescents (MINI-KID) [22], prior treatment with at least two SSRIs at an adequate dose, stable regimen of medications for three weeks prior to treatment, with no changes throughout the course of treatment. Exclusion criteria were: epilepsy or medical history of seizures, conductive ferromagnetic or other magnetic sensitive metals implanted in the head or within 30 cm of the treatment coil, implanted device that is activated or controlled in any way by physiological signals, implanted medication pumps, intracardiac lines (even if removed), active substance use disorder, treatment with any medication that could lower the threshold for seizures, usage of benzodiazepines either as prescribed drug or illegal use, or any condition that seriously increases the risk of non-compliance or loss of follow-up.

### rTMS treatment parameters

The clinical procedure followed during the intervention was identical to that used for adults at the brain stimulation unit at Uppsala University Hospital. Initially, the resting motor threshold was determined by finding the minimal intensity that produces a visually observed motor twitch in the contralateral distal wrist muscles, using single pulses over the left motor cortex [23]. The rTMS was delivered with a powerMAG research ppTMS stimulator (Mag & More), and a figure-of-eight coil, PMD70-pCool (Mag & More). The research participants received 1 Hz rTMS with daily sessions on consecutive week days for 4 weeks. The treatment was extended to 6 weeks for patients that responded with ≥ 20% reduction on MADRS. The magnetic pulses were applied at 120% of the resting motor threshold with a figure-of-eight coil at a 45 degree angle towards the midline. The 1 Hz rTMS protocol consists of 6 trains of 1-min duration separated by 30-sec inter-train “off” periods. It was applied over the right DLPFC at a target corresponding roughly to the F4 site according to the 10–20 system, using a structured and validated way of locating the treatment target with measuring tape [24]. The total duration of one 1 Hz rTMS session is 8 min 30 s [9].

### Clinical assessment and outcomes

Before, and four and six weeks after treatment start, a clinical psychiatric evaluation was conducted. Patients were evaluated regarding side-effects daily and regarding psychiatric well-being on a weekly basis. Treatment resistance was assessed using the Maudsley Staging Method for treatment resistant depression [25]. The positive and negative expectation associated with treatment was assessed using the Stanford Expectations of Treatment Scale (SETS) [26].

The primary outcomes of this study are measures of study feasibility as measured by inclusion- and attrition rate as well as safety and tolerability. Secondary measures are changes in the severity of depression symptoms measured by self- and clinician rated Montgomery-Asberg Depression Rating (MADRS) [27]. Additional secondary measures of symptoms and function were also assessed (see Supplementary Methods and Results).

### Statistical methods

All analyses were performed using R Statistical Software v4.4.1 [28].

Due to a small sample size, normality of the data cannot reliably be assumed and thus the non-parametric Wilcoxon signed-rank test was used for statistical hypothesis testing of paired observations using the *rstatix* package [29, 30], *p*-values and effect size *r* were computed for each comparison. For pairwise correlations Spearman’s rank correlation was used and Spearman rho as well as *p*-value were reported.

For repeated measures, linear mixed-effects models were used via restricted maximum likelihood (REML) criterion of convergence [31]. Outcome measure was chosen as dependent variable, time (treatment day) as fixed effect and participant-id was treated as a random effect. Fixed-effects parameter estimates β as well as standard error (SE) of the estimate were reported. The *lmerTest* package was used to estimate *p*-values using Satterthwaite’s method [32]. Results were treated as statistically significant when *p* < 0.05.

Results were plotted with the *ggplot2* or *ggpubr* package [33, 34].

## Results

### Primary outcomes

**Fig. 1:**
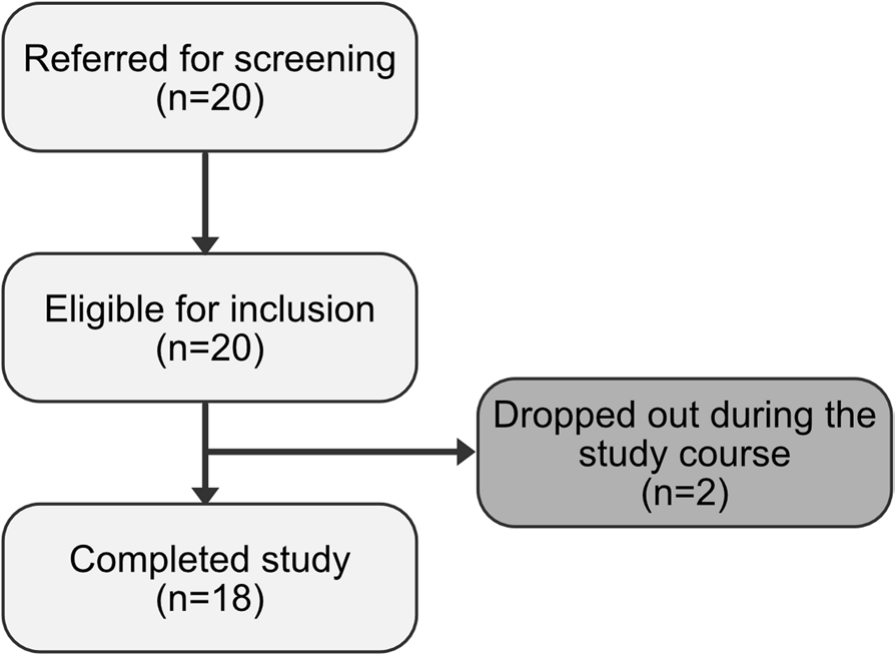
Participant enrollment. Note: Flow chart of participant enrollment

Between January 2022 and October 2023 a total of 20 patients were included in this open-label feasibility study, thus yielding an inclusion rate of 0.9 patients/month (Table 1; Fig. 1).

**Table 1:** Patient demographics and baseline clinical characteristics

Baseline characteristic	
Sex, n (male/female/other)	3/16/1
Age, median (range)	17 (13–18)
Medications*, n	
SSRI	13
Other antidepressants	5
Mood stabilizers	3
Antipsychotic	1
Antihistamine	10 (2 as needed, 7 regular interval, 1 both)
Stimulant	3
	Median (IQR), n
MADRS-S	34 (10), 19
MADRS-M	35 (7.5), 20
QIDS-SR	20 (6), 19
EQ-5D VAS/NRS	20 (12.5), 17
CGAS	45 (14), 20
RCADS-P depression	19 (3.25), 18
RCADS-P anxiety	46.5 (22.75), 18
RCADS-C depression	20 (4), 19
RCADS-C anxiety	46 (19), 19
AS-18 mania	10 (4), 19
AS-18 depression	22 (10), 19
CAPE-15	20 (6), 19
SETS positive	3.8 (2), 20
SETS negative	2 (2.5), 20
CPRS memory question	2 (2), 19
MSM	8 (1.75), 20
AUDIT	0 (1), 20
DUDIT	0 (0), 20

*N* = 20. Abbreviations: *SSRI * selective serotonin reuptake inhibitor, *MADRS* Montgomery-Asberg Depression Rating Scale, *S* self-rated, *M* clinician rated, *QIDS-SR * Quick Inventory of Depressive Symptomatology– self-rated, *EQ-5D VAS/NRS* EQ-5D Visual Analogue Scale/Numerical Rating Scale, *CGAS* Children’s Global Assessment Scale, *RCADS* Revised Child Anxiety and Depression Scale, *P* Parent rated, *C* Child self-rated; AS-18 = Affective Self-Rating Scale, *CAPE-15* Community Assessment of Psychic Experiences, *SETS* Stanford Expectations of Treatment Scale, *CPRS* Comprehensive Psychopathological Rating Scale, *MSM* Maudsley Staging Method, *AUDIT* Alcohol Use Disorders Identification Test, *DUDIT * Drug Use Disorders Identification Test

*see Supplementary Materials and Results for a detailed list of medications

All patients had an ongoing depressive episode and all but one unipolar disorder (*n* = 19) while one had a bipolar disorder (type 2). Two patients in total dropped out during the follow-up period, one due to acquisition of a job which made participation impossible and one that did not show up for planned visits after screening and inclusion. The drop-out resulted in an attrition rate of 0.10, and 18 patients in total completed the study.

**Fig. 2:**
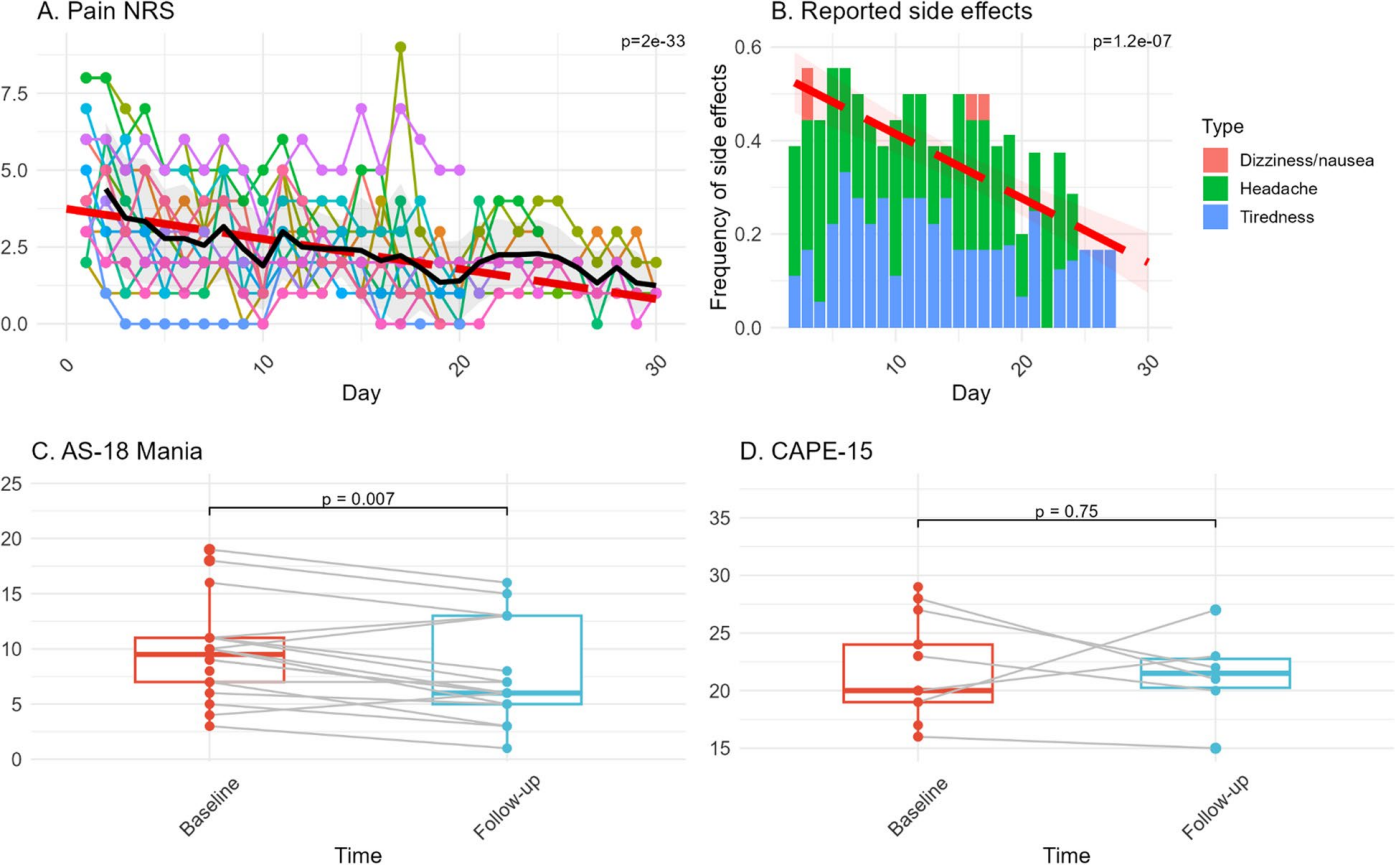
Adverse effects.Note: Analysis of adverse events. Individual trajectories of pain numerical rating scale (NRS) with mean (solid black line), standard deviation (shaded grey area) and estimated slope and intercept of fixed effect ‘Day’ (red dashed line) (**A**). Stacked bar plot with frequency of reported side effects per treatment session, with linear trend predicted by linear model (red dashed line), confidence interval (shaded red area) (**B**). Paired measurements of Affective Self-Rating Scale Manic Symptoms (AS-18 Mania) (**C**) and Community Assessment of Psychic Experiences (CAPE-15) (**D**) before and after treatment

Scalp pain was quantified using a pain numerical rating scale (NRS), ranging from 1 to 10 after the first and sixth train of rTMS, the maximum of these measurements for each session and patient was analyzed using a linear mixed model. The resulting model implies a significant decrease of scalp pain over time (β=−0.10, SE = 0.007, *p* = 2e-16***) (Fig. 2 A).

Headache was the most common side effect that was reported in connection with treatment (91/426 sessions, 21%), followed by tiredness (75/426 sessions, 18%) while dizziness/nausea was only reported in 4 sessions (Fig. 2B). Like for scalp pain, there was a significant reduction in the frequency of reported headache, tiredness or dizziness/nausea over time (linear model, β=−0.014, *p* = 1.24e-07***). One patient reported increased irritability after the 3rd session and one patient reported increased anxiety and suicidal ideation after the 23rd session, which necessitated hospitalization. One patient reported fainting during the night after the 18th treatment and after the 24th treatment the same patient conducted a suicide attempt by intoxication, this event was classified as severe and as probably related natural disease progression, but the possibility of relation to treatment could not be ruled out.

There was no increase in symptoms of mania as measured by the AS-18 Mania score, rather a decrease over time (Median_baseline_=10, Median_follow-up_ = 6, *p* = 0.007**, *r* = 0.661) (Fig. 2 C) and no effect of treatment on psychotic symptoms reported in CAPE-15 could be observed (Median_baseline_=20, Median_follow-up_ = 22, *p* = 0.75^ns^, *r* = 0.171) (Fig. 2D).

## Secondary outcomes

**Fig. 3:**
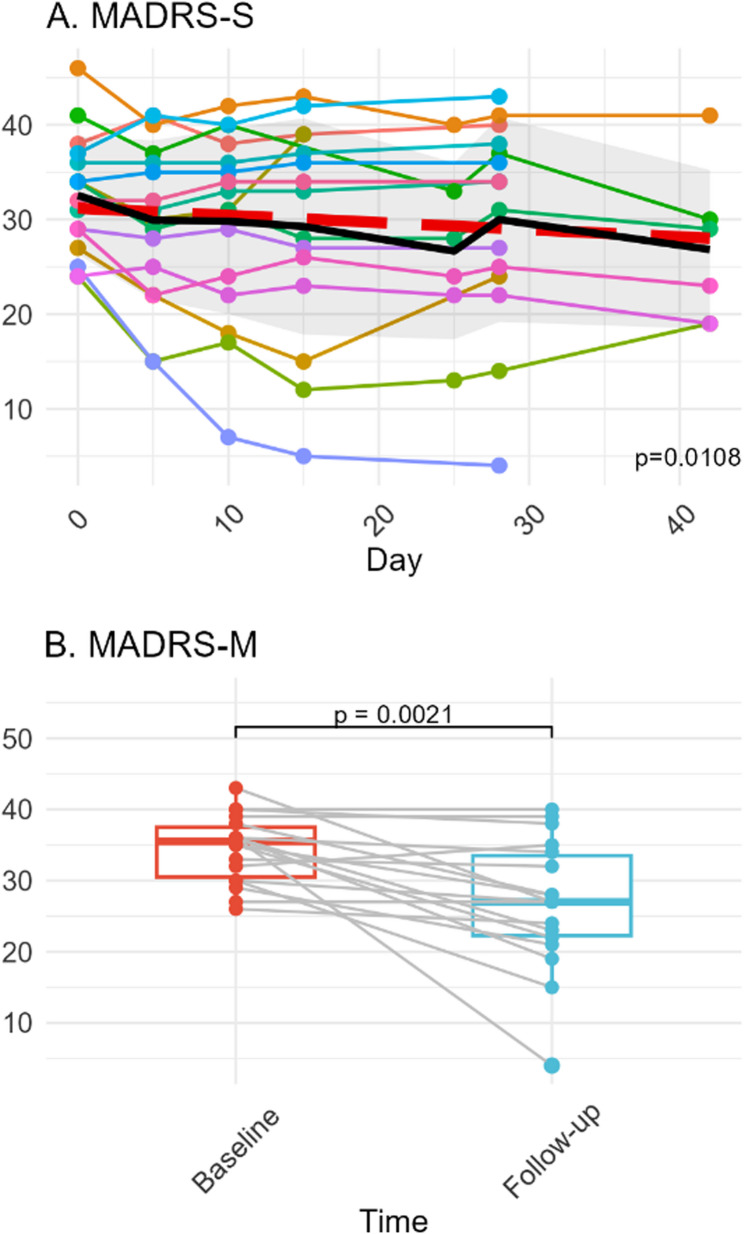
Depressive symptom outcome measures. Note. Patient rated MADRS-S (**A**) with individual trajectories with mean (solid black line), standard deviation (shaded grey area) and estimated slope and intercept of fixed effect ‘Day’ (red dashed line). Clinician rated paired MADRS-M (**B**) measurements at baseline and follow-up. Abbreviations: MADRS = Montgomery-Asberg Depression Rating Scale, S = self-rated, M = clinician rated.

A significant effect of time could be observed in MADRS-S (β=−0.076, SE = 0.029, *p* = 0.011*) (Fig. 3 A). Clinician rated MADRS-M decreased (Median_baseline_=35, Median_follow-up_ = 27, *p* = 0.002**, *r* = 0.762) from baseline to follow-up (Fig. 3B). Although significant decreases in MADRS-S and MADRS-M were observed, only 1 of 18 patients reached response criteria (> 50% reduction of MADRS), the same patient also reached remission (< 10p MADRS). Additional clinical outcome measures are reported in Supplementary Methods and Results. One patient was diagnosed with bipolar disorder type 2 and excluding this individual in the analysis did not significantly alter the results.

### Expectations on outcome

There was no detectable correlation between positive expectation (Stanford Expectations of Treatment Scale, SETS positive) on treatment outcome (MADRS-S) (Spearman rank correlation, rho = −0.2, *p* = 0.430^ns^). Neither were there any correlation between negative expectation (SETS negative) and frequency of reported side effects (Spearman rank correlation, rho = −0.23, *p* = 0.368^ns^).

## Discussion

A significant proportion of adolescents suffering from depression do not respond to conventional treatment with psychotherapy or serotonin reuptake inhibitors, leading to prolonged suffering, loss of quality of life and functioning. This was the first-ever open-label pilot study to investigate the feasibility, tolerability and potential efficacy of using low-frequency (1 Hz) rTMS over the right DLPFC for TRD in adolescents. Our findings show that while 1 Hz rTMS was feasible and generally well-tolerated, and the decrease in depressive symptoms was significant, only one patient reached remission.

Within the given timeframe and population, the inclusion rate of 0.9 patients per month and attrition rate of 0.10 demonstrate feasibility. The study maintained high participant retention with 18 of the 20 enrolled participants completing the full treatment course of at least 4 weeks of treatment.

Although a significant decrease in depressive symptoms was observed (MADRS-S and MADRS-M scores) along with an increase in functioning (EQ-5D and CGAS, see Supplementary Methods and Results and Figure S1), only one patient reached response and remission indicating a modest antidepressant effect in the current population. This is in line with previous studies of rTMS in the adolescent population, for instance a large randomized sham-controlled study of high-frequency left DLPFC rTMS failed to detect significant decrease in symptoms, suggesting that rTMS might not be as effective as in the adult population [13]. Surprisingly, prior expectations of treatment outcome (SETS positive and negative) did not correlate with change in self-rated depressive symptoms (MADRS-S) or with tendency to report side effects, which suggest that the placebo- and nocebo effects are not important factors in explaining the variance in outcome.

The side effects reported were generally mild and tended to decrease over time. However, a few serious events occurred, including a suicide attempt during the course of treatment which probably is related to the underlying disease but possibly also could be related to the treatment itself. Such events should warrant caution and proper monitoring in future studies. No patients developed seizures during the trial, which has been reported as a rare but serious adverse event in previous studies [10].

While electroconvulsive therapy constitute an effective second-line treatment of TRD in both adult and adolescent populations, it is sometimes associated with adverse effects such as memory impairment, tardive seizures and complications due to general anesthesia, that might deter patients or guardians from consenting to treatment [35]. Even though only one patient reached response in our current study, there were still a statistically significant decrease in depressive symptoms. Further, a six-point reduction in MADRS-M has been proposed to be a clinically meaningful symptom decrease and in our current study there was a median reduction of eight points, indicating a potential clinical value [36]. In the light of lacking treatment options in the treatment resistant adolescent population this indicates that low-frequency rTMS is still a promising treatment that should be further evaluated, preferably in a larger RCT for patients that are not eligible for treatment with electroconvulsive therapy. Future studies of 1 Hz rTMS for adolescent TRD should focus on larger samples sizes with a proper sham-control arm. The clinical rating and subsequent data analysis should maintain blinding to treatment, which was not the case in this study due to the lack of control group. Another potential factor to consider boosting efficacy, is to increase the number of pulses, as more than 1200 pulses in low-frequency protocols has been associated to better effect in a previous meta-analysis [37]. Although rTMS still holds promise as an alternative antidepressant treatment in adolescents, more robust evidence is needed to fully establish its role in the treatment arsenal.

### Limitations

This study suffers several limitations, mostly given the pilot study design. The sample size is small and lack a sham control group which precludes clear conclusions regarding possible antidepressant effects and safety profile. There was no blinding in the study which could introduce bias. There was also a variability in the number of treatments that each participant received, which could have affected the outcome. Further, there was no follow-up of the durability of the effect.

## Conclusions

This study provides preliminary findings suggesting that 1 Hz rTMS is feasible, generally safe and tolerable in adolescents with TRD. The effects on depressive symptoms and functioning were moderate but promising. The study highlights the necessity of carrying out larger and well-controlled studies to elucidate the role of rTMS in this difficult to treat population.

## Supplementary Information

Supplementary Material 1.

Supplementary Material 2.

Supplementary Material 3.

## Data Availability

The datasets used and/or analysed during the current study are available from the corresponding author on reasonable request.
