# Supplementary material for: Low-frequency repetitive transcranial magnetic stimulation for adolescent treatment resistant depression - a feasibility study
Source: BMC Psychiatry. 2025 Jul 3;25:679. doi: 10.1186/s12888-025-07115-5 (PMC12231907; doi:10.1186/s12888-025-07115-5)
Supplement: Supplementary file 1 — Supplementary Material 1. [file 12888_2025_7115_MOESM1_ESM.docx]

Supplementary methods and results

Methods

Additional secondary measures include the overall level of functioning estimated by the clinician-rated Children’s Global Assessment Scale (CGAS) [1] and the following self- or parent reported clinical measures: Quick Inventory of Depressive Symptomatology- Self Report (QIDS-SR) [2], EQ-5D [3], Alcohol Use Disorders Identification Test (AUDIT) [4], the Drug Use Disorders Identification Test (DUDIT) [5], Affective Self Rating Scale (AS-18) [6], Child Anxiety and Depression Scale (RCADS) [7], Community Assessment of Psychic Experiences (CAPE-15) [8].

Results

Medications

The study participants were prescribed a range of psychotropic medications: antidepressants agomelatine (n=1), escitalopram (n=4), fluoxetine (n=4), mirtazapine (n=3), sertraline (n=5) and vortioxetine (n=1); anxiolytic antihistamines alimemazine (n=2), hydroxyzine (n=1) and promethazine (n=7); stimulants methylphenidate (n=1), lisdexamfetamine (n=1) and guanfacine (n=1); mood stabilizers lithium (n=2), lamotrigine (n=1); sleep aids propiomazine (n=3) and melatonin (n=13); antipsychotic quetiapine (n=1).

Additional Clinical Outcomes

A significant effect of time could be observed in both repeatedly measured patient rated outcomes (Figure S1), AS-18 Depression (β=-0.123, SE=0.027, p=<0.001***) and EQ-5D VAS (β=0.184, SE=0.066, p=0.006**). Pairwise comparison of baseline versus follow-up measurements yielded large and significant decreases in ratings of QIDS-SR (Median_baseline_=20, Median_follow-up_=17, p=0.011*, r=0.634) and RCADS-C Depression (Median_baseline_=20, Median_follow-up_=17, p=0.008**, r=0.667) but not RCADS-C Anxiety (Median_baseline_=46, Median_follow-up_=46, p=0.078, r=0.420). Both the depression (Median_baseline_=19, Median_follow-up_=14, p=0.001**, r=0.817) and anxiety (Median_baseline_=46.5, Median_follow-up_=32.5, p=0.002**, r=0.873) scores of the parent rated RCADS-P were decreased at follow-up. Clinician rated CGAS increased (Median_baseline_=45, Median_follow-up_=55, p=0.016**, r=0.614) from baseline to follow-up.

References

1. Shaffer D, Gould MS, Brasic J, Ambrosini P, Fisher P, Bird H, et al. A children’s global assessment scale (CGAS). Arch Gen Psychiatry. 1983;40:1228–31.

2. Bernstein IH, Rush AJ, Trivedi MH, Hughes CW, Macleod L, Witte BP, et al. Psychometric properties of the Quick Inventory of Depressive Symptomatology in adolescents. Int J Methods Psychiatr Res. 2010;19:185–94.

3. EuroQol Group. EuroQol--a new facility for the measurement of health-related quality of life. Health Policy Amst Neth. 1990;16:199–208.

4. Bohn MJ, Babor TF, Kranzler HR. The Alcohol Use Disorders Identification Test (AUDIT): validation of a screening instrument for use in medical settings. J Stud Alcohol. 1995;56:423–32.

5. Berman AH, Bergman H, Palmstierna T, Schlyter F. Evaluation of the Drug Use Disorders Identification Test (DUDIT) in Criminal Justice and Detoxification Settings and in a Swedish Population Sample. Eur Addict Res. 2005;11:22–31.

6. Adler M, Liberg B, Andersson S, Isacsson G, Hetta J. Development and validation of the Affective Self Rating Scale for manic, depressive, and mixed affective states. Nord J Psychiatry. 2008;62:130–5.

7. Chorpita BF, Yim L, Moffitt C, Umemoto LA, Francis SE. Assessment of symptoms of DSM-IV anxiety and depression in children: a revised child anxiety and depression scale. Behav Res Ther. 2000;38:835–55.

8. Capra C, Kavanagh DJ, Hides L, Scott J. Brief screening for psychosis-like experiences. Schizophr Res. 2013;149:104–7.
