# Supplementary figures and images for: Low-frequency repetitive transcranial magnetic stimulation for adolescent treatment resistant depression - a feasibility study
Source: BMC Psychiatry. 2025 Jul 3;25:679. doi: 10.1186/s12888-025-07115-5 (PMC12231907; doi:10.1186/s12888-025-07115-5)

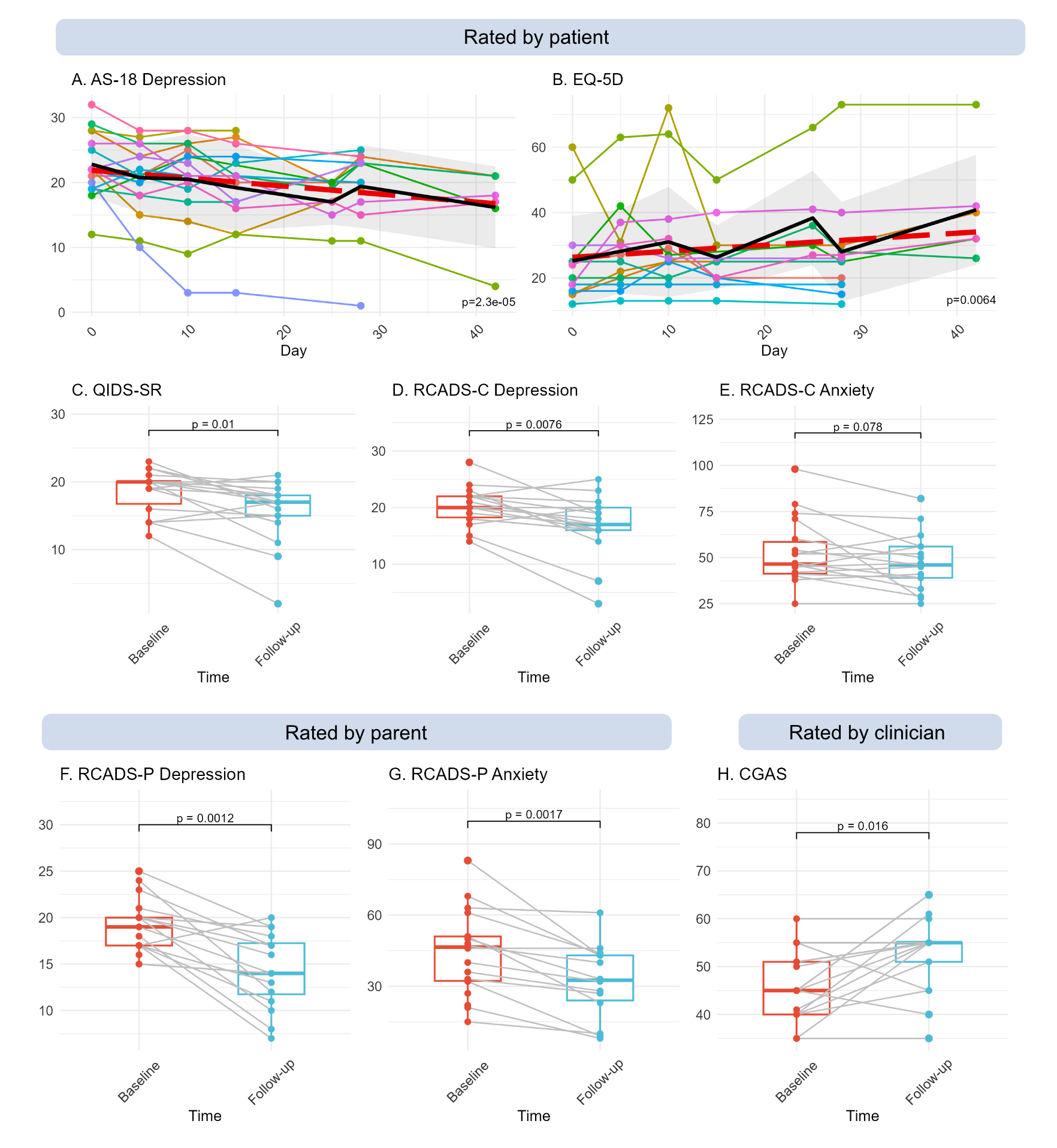

Supplement: Supplementary file 2 — Supplementary Material 2. [file 12888_2025_7115_MOESM2_ESM.tiff]
