# Supplementary material for: Low-frequency repetitive transcranial magnetic stimulation for adolescent treatment resistant depression - a feasibility study
Source: BMC Psychiatry. 2025 Jul 3;25:679. doi: 10.1186/s12888-025-07115-5 (PMC12231907; doi:10.1186/s12888-025-07115-5)
Supplement: Supplementary file 3 — Supplementary Material 3. [file 12888_2025_7115_MOESM3_ESM.docx]

Figure S1. Additional secondary outcome measures

Note. Summary of secondary outcomes rated by the patient (A-E), parent (F, G) and clinician (H). Panels A-B display individual trajectories with mean (solid black line), standard deviation (shaded grey area) and estimated slope and intercept of fixed effect ‘Day’ (red dashed line). Abbreviations: QIDS-SR = Quick Inventory of Depressive Symptomatology – self-rated; EQ-5D = EQ-5D Visual Analogue Scale; CGAS = Children's Global Assessment Scale; RCADS = Revised Child Anxiety and Depression Scale, P = Parent rated, C = Child self-rated; AS-18 = Affective Self-Rating Scale.
